# Targeting lipid metabolism in acute myeloid leukemia: biological insights and therapeutic opportunities

**DOI:** 10.1038/s41375-025-02645-z

**Published:** 2025-05-22

**Authors:** Vilma Dembitz, Sophie C. James, Paolo Gallipoli

**Affiliations:** 1 Department of Physiology and Croatian Institute for Brain Research, University of Zagreb School of Medicine, Zagreb, Croatia; 2 Centre for Haemato-Oncology, Barts Cancer Institute, Queen Mary University of London, London, UK

**Keywords:** Translational research, Cancer metabolism

## Abstract

Metabolic rewiring is a hallmark of malignant transformation in leukemic cells and the potential offered by its therapeutic targeting has garnered significant attention. The development of clinically relevant metabolic targeted therapies in acute myeloid leukemia (AML) has mostly focused on targeting mitochondrial energy production, but progress has been hampered by generalized toxicities. An alternative strategy is to shift the focus from targeting energy production to targeting more specialized metabolic functions, such as energy storage, the regulation of oxidative stress and availability of cofactors needed for the function of specific metabolic reactions. Lipid metabolism plays a role in many of these metabolic functions and its importance in AML maintenance and response to therapy is being increasingly recognized but needs to be adequately interpreted in the context of its interaction with the microenvironment, particularly the adipose niche. In this review, we provide an overview of our current understanding of AML cellular metabolic dependencies on fatty acid and lipid metabolism and discuss their relevance in the context of functional interactions with adipocytes. We highlight unresolved questions about how to best target lipid metabolism and suggest approaches needed to fully understand the interplay between malignant cells and their niche in the context of metabolic dependencies.

## Introduction

Over the last two decades, improved understanding of acute myeloid leukemia (AML) genetics and biology combined with a strong focus on development of targeted therapies has led to several novel compounds being approved for AML treatment. Despite these improvements, the long-term prognosis for the majority of AML patients still remains unfavorable [1, 2]. Although AML has fewer mutations compared to many other cancers, one of the key challenges in developing effective treatments is its significant clonal heterogeneity [3]. As a result, therapeutic strategies that primarily target driver mutations are likely to produce only partial or short-lived responses, even when used in combination. Moreover, the recurrent mutations for which specific inhibitors have been successfully developed are present only in patient subsets, while many other recurrent AML mutations are not ideal therapeutic targets for small molecule design, being either loss-of-function mutations or mutations in transcription factors [3]. Thus to achieve significant improvements in patient survival, it is essential to identify specific oncogenic molecular vulnerabilities that are both therapeutically actionable and ideally present across various genetic subgroups. Indeed, the most successful novel therapy approved in AML over the last decade has been venetoclax, an inhibitor of the antiapoptotic protein BCL-2, which is a common dependency across multiple AML subtypes and other cancer types.

Cancer-specific metabolic changes have emerged as therapeutically actionable vulnerabilities present across different types of malignancies. Rewired metabolism is considered a hallmark of malignant transformation and an increasing body of research is unraveling how cancer cells rely on pathways that control both energy and biomass production [4]. In AML, metabolic research initially focused on mitochondria function and its role in energy production through cellular respiration. Although AML cells, like many malignant cells, undergo significant glycolysis even in aerobic conditions, the so called Warburg effect, they also display a strong dependency on mitochondrial metabolism and oxidative phosphorylation for their survival [5–7]. However, since mitochondrial metabolism is essential for all active cells, targeting it pharmacologically will require further optimization as all current approaches have resulted in either lack of clinical effectiveness [8] or generalized toxicity [9].

An alternative metabolic strategy would be to target more specialized pathways, such as lipid metabolism. Modulating these pathways can induce subtle metabolic changes, such as alterations in cofactor availability for abnormal signaling and transcription [10], while also affecting redox balance [11] and cellular stress responses [12] – key oncogenic features commonly observed in leukemic cells. The role of lipid metabolism in AML has long been recognized, with prior studies demonstrating a metabolic dependency on cholesterol homeostasis, thereby underscoring its potential as a therapeutic target. This resulted in clinical trials assessing the potential benefit of adding statins to idarubicin and cytarabine treatment regimens. Although this protocol was safe in early phase trials, in larger trials the response rates were not consistent with a positive study [13, 14]. Nevertheless, a recent study showed increased efficacy of venetoclax based regimens in combination with statins in different hematologic malignancies [15] leading to successful completion of Phase I clinical trial of adding pitavastatin to venetoclax therapy in AML and chronic lymphocytic leukemia [16]. This together with accumulating preclinical evidence on the role of lipid metabolism in AML has reignited interest in translational research focusing on different facets of lipid metabolism in AML.

Indeed, both the cellular compartment [17] and the plasma [18, 19] of AML patients have a distinct lipid signature in comparison with healthy controls and several reports observed differences in lipid species which correlate with specific cytogenetic and prognostic groups [20]. Several preclinical studies have demonstrated that lipid metabolism is crucial for the survival and function of leukemic cells [7, 11, 12, 21–23]. Therefore gaining a deeper understanding of its role in AML holds significant potential for discovering new therapeutic strategies. In this review, we will summarize the current knowledge on the involvement of lipid metabolism in AML, emphasizing both intrinsic cellular alterations and niche-mediated modulation of this metabolic pathway with a specific focus on the role of adipocytic niche. Additionally, we will identify areas for future research and potential clinical applications.

## Lipid metabolism overview

From a chemical point of view, lipids are a compound group that was historically defined as being non soluble in water but soluble in organic solvents. Clearly, such chemical definition is very loose and the term “lipids” therefore comprises structurally and functionally diverse molecules that are traditionally divided into fatty acids (FA), steroids, acylglycerols, phosphoglycerols (also known as phospholipids), and sphingolipids.

Cells obtain molecules needed for lipid generation through uptake or de novo synthesis. Fatty acids (FAs), which serve as the backbone for most lipid groups, are water-insoluble molecules that require closely regulated transport across the cell membrane. This transport is facilitated by several transporter proteins, including fatty acid binding proteins (FABP), fatty acid transport proteins (FATP), fatty acid translocase (FAT/CD36), and caveolin-1 [24–26]. Interestingly, CD36 was initially used as an immature myeloid or erythroid marker [27], before its role in FA transport was associated with cancer progression and metastasis [28–30]. Most cells preferentially use extracellular uptake as a method for replenishing their FA pools and FA synthesis (FAS) predominantly occurs in hepatocytes and adipocytes. However, upregulation of FAS is a characteristic of a malignantly transformed cell [31, 32]. Several inhibitors of fatty acid synthesis enzymes are currently undergoing clinical development for treating both solid cancer and metabolic disorders (Table 1). If proven successful, these inhibitors may offer promising opportunities for drug repurposing for the treatment of hematologic malignancies.

**Table 1:** Fatty acid synthesis inhibitors in clinical development.

Target	Inhibitor	Condition
ACLY	Bempedoic acid	Hyperlipidemia (FDA, EMA approved) [124]
ACC	Clesacostat (PF-05221304)	Non-alcoholic steatohepatitis (Phase II) [125]
ACC	Firsocostat (GS-0976)	Non-alcoholic steatohepatitis (Phase II) [126]
FASN	Denifanstat (TVB-2640)	Non-alcoholic steatohepatitis (Phase III) [127] Metastatic solid tumors (Phase II) [128]
FASN	ASC40	Glioblastoma (Phase III) [129]
SCD1	MTI-301	Metastatic or Unresectable and Refractory Solid Cancers (Phase I) [79]

**Fig. 1:**
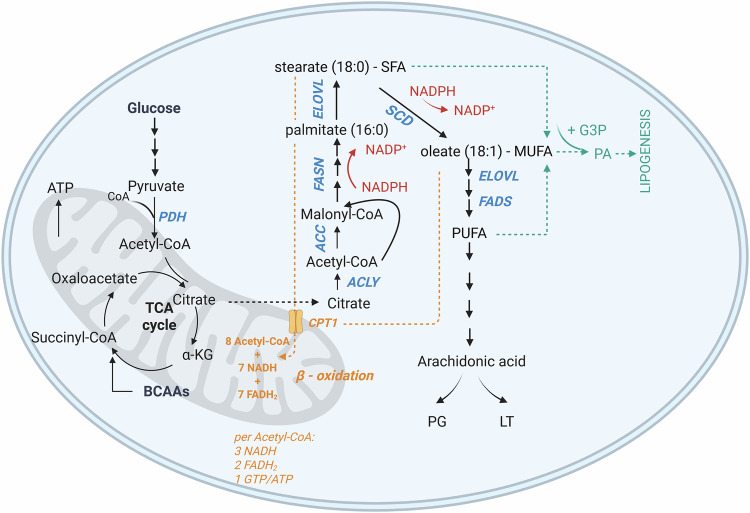
Fatty acid synthesis and oxidation. PDH pyruvate dehydrogenase, ACLY ATP citrate lyase, ACC Acetyl-CoA carboxylase, CPT1 Carnitine palmitoyltransferase I, FASN fatty acid synthetase, ELOVL fatty acid elongase, SCD stearoyl-CoA desaturase, FADS fatty acid desaturase, α-KG α-ketoglutarate, TCA cycle tricarboxylic acid cycle, PG prostaglandins, LT leukotrienes, SFA saturated fatty acids, MUFA monounsaturated fatty acids, PUFA polyunsaturated fatty acids, G3P glycerol-3-phosphate, PA phosphatidic acid. Figure was generated using Biorender.com.

The conversion of pyruvate, the product of glycolysis, to acetyl-CoA can initiate the tricarboxylic acid (TCA) cycle when acetyl-CoA is bound to oxalacetate to form citrate. Alternatively, when citrate is produced in surplus, it is transferred to the cytoplasm where it is again cleaved into acetyl-CoA and oxaloacetate through the activity of ATP citrate lyase (ACLY). Acetyl-CoA in the cytoplasm can then serve as a precursor for acetyl-CoA carboxylases (ACC). ACC catalyzes the conversion of acetyl-CoA to malonyl-CoA, marking the first committed step in FAS (Fig. 1). Malonyl-CoA and acetyl-CoA are then combined into the saturated fatty acid (SFA) palmitate (C16:0) by the enzyme fatty acid synthase (FASN) [33]. ACLY, ACC, and FASN have long been recognized as promising therapeutic targets in solid tumors [33], but their roles in AML remain less well understood. Low expression of ACLY has been linked to a potentially favorable prognosis in AML [34], while emerging evidence suggests that FASN may be essential for leukemogenesis [35]. These findings support the pro-oncogenic roles of these enzymes, as previously observed in solid cancers [33], and underscore their potential in the development of metabolism-based therapies. However, unlike ACLY and FASN, stabilization of ACC has been shown to impair leukemogenesis, with ACC1 appearing to act as a suppressor of AML progression [36]. This indicates a more complex role for ACC in AML and may limit the therapeutic value of targeting this enzyme directly.

Palmitate can subsequently be activated by acyl-CoA synthase, producing palmitoyl-CoA which can translocate to mitochondria for degradation in the process of β-oxidation, or desaturated by stearoyl-CoA desaturase (SCD), producing a monounsaturated fatty acid (MUFA), or elongated into stearate (C18:0) by the activity of FA elongases (ELOVL) (Fig. 1). Desaturation and elongation steps can be re-iterated until a spectrum of FA with variable length and saturation level is produced [31].

Fatty acid desaturation is especially intriguing in the cancer context. The main FA desaturase, SCD, transforms SFA palmitate (C16:0) and stearate (C18:0) into MUFA palmitoleate (C16:1) and oleate (C18:1). SCD activity has been implicated in cancer cell survival [23, 37, 38], and its overexpression has been identified in several malignancies, often in association with adverse prognosis [23, 39–41]. MUFA production by SCD can be circumvented in the malignant cell by the activity of other fatty acid desaturases (FADS), namely FADS2 [42]. FADS1 and FADS2 are downstream desaturases that in physiological conditions introduce subsequent unsaturated bonds in MUFA produced by SCD and are responsible for polyunsaturated FA (PUFA) production. Same as SCD, both FADS1 and FADS2 are adversely prognostic in solid tumors and have thus been highlighted as potential therapeutic targets [43, 44]. When discussing the involvement of PUFA in the cancer cell one must keep in mind that the majority of PUFA can be synthesized in humans, but linoleic and alpha-linoleic acids are essential and need to be acquired through the diet [33]. FADS2 converts them into arachidonic acid and, interestingly, palmitate can compete with linoleic and alpha-linoleic acids in this reaction [45], which could explain the ability of FADS2 to compensate for the lack of SCD activity in cancer cells [42].

FA length and saturation level direct their role in cellular functions. Both SFA and PUFA have been described as inducers of oxidative stress [46, 47] that can govern apoptotic and ferroptotic forms of cell death [48], while MUFA decrease cancer cell sensitivity to cell death in response to lipid reactive oxygen species (ROS) accumulation [49]. Saturation levels are crucial determinants of both membrane fluidity and endoplasmic reticulum stress, the main intrinsic factor controlling the integrated stress response [50]. FA length and saturation also governs their incorporation into more complex lipids and helps determine the diversity of phospholipids and sphingolipids and their involvement in signal transduction. The potential involvement of sphingolipids in AML aggressiveness is particularly intriguing as AML patients display distinct plasma sphingolipid profiles in comparison to healthy individuals [51] and their sphingolipidomic subtype appears to be linked with AML prognosis [52]. In contrast to healthy cells, in malignant cells sphingolipids are crucial regulators of cell death and apoptosis is marked by the buildup of ceramide and dihydroceramide [53]. These observations are particularly interesting in the light of novel treatment approaches in AML focused on targeting the apoptotic machinery [54]. Furthermore, phospholipid signaling which is downstream of growth factor receptors is a field of cancer biology on its own [55]. However, the complexity of lipids’ role in signaling and cell fate regulation goes beyond the scope of this review and we refer readers to review articles that focus specifically on this area [56, 57].

Finally, either absorbed from the surroundings or synthetized de novo, FA can serve as an energy source through the mitochondrial process of β-oxidation or fatty acid oxidation (FAO). Carnitine acetyl transferases (CPT1 on the outer and CPT2 on the inner mitochondrial membrane) shuttle FA from the cytoplasm into the mitochondria where it undergoes sequential oxidation steps with NAD^+^ and FAD serving as electron acceptors. Palmitate oxidation (Fig. 1) in the mitochondrion generates 8 molecules of acetyl-CoA, 7 NADH, and 7 FADH2. After NADH and FADH2 enter the electron transport chain (ETC), and acetyl-CoA enters the TCA cycle, the process produces a total of 106 ATP molecules. This illustrates the substantial energy yield from fatty acid oxidation (FAO) [58, 59]. As mentioned previously, excess acetyl-CoA entering the TCA cycle can lead to a surplus of citrate. This citrate is then transported into the cytoplasm, where it serves as a precursor for the activity of malic enzyme and isocitrate dehydrogenase, ultimately producing NADPH. The balance of oxidized and reduced forms of NADP could explain the seemingly inefficient coupling of FAS and FAO. FAS is a major NADPH consumer while FAO is a great NADPH producer and the ratio of NADPH/NADP^+^ links FA metabolism to the pentose phosphate pathway as well as serine metabolism and one carbon metabolism, providing further potential insights into their interplay in cancer cells [4].

The importance of lipid metabolism in cancer has been highlighted for more than 50 years by pathology reports describing accumulation of lipid droplets in several subtypes of malignant cells [60]. However, the puzzle of interactions between lipid uptake, synthesis and storage on one side and catabolism on the other is only starting to be assembled both in solid and hematologic malignancies.

## Fatty acid oxidation is a functional dependency of the leukemic stem cell

Metabolic differences between leukemic stem cells (LSC) and their healthy counterparts opens a potential therapeutic window for specific targeting of malignant cells. Hematopoietic stem cells (HSC) generate energy predominantly through glycolysis [61]. Apart from adaptation to the hypoxic bone marrow (BM) niche where HSCs reside, glycolysis is also associated with lower ROS production and decreased oxidative damage. This is required for maintaining HSCs in a quiescent state and sustaining their self-renewal [62, 63]. An increase in mitochondrial activity can direct HSC differentiation [64] in response to stressors. For example, switching energy production from glycolysis to FAO governs HSC expansion and mature leukocyte production in response to bacterial infection [65]. In contrast to HSCs, LSCs rely on oxidative phosphorylation (OxPhos) for energy production and cannot utilize glycolysis when mitochondrial respiration is inhibited. However, this dependence on oxidative metabolism is associated with lower overall levels of oxidative activity, lactate production, ATP content, and ROS levels, reflecting their dormant state [6]. OxPhos in de novo LSCs appears to be predominantly fueled by amino acids, but in relapse it switches to FAO, leading to a decreased sensitivity to venetoclax based regimens [21, 66]. The observation that sensitivity to venetoclax depends on FAO is further strengthened by findings in relapsed/refractory patients that venetoclax resistance correlates with expression of an OxPhos signature and CD36 [67]. The association between FAO and relapse extends beyond venetoclax based regimens. The cytarabine resistant cell population is not enriched for either LSCs as defined by immunophenotype or transcriptomic stem cell signatures or leukemia-initiating cells, but displays higher levels of ROS and OxPhos fueled by FAO and increased expression of CD36 [7]. Comparably, mice treated with a chemotherapy protocol mimicking the 3 + 7 anthracycline/cytarabine protocol had an enriched OxPhos and FA metabolism signature in leukemic cells at disease nadir, further implying association with chemoresistance [68]. Indeed, expression of CPT1A, which transports FA into the mitochondria and regulates the entry point for FAO, is negatively prognostic in AML [69]. Furthermore, its pharmacological inhibition has antileukemic effects in vitro, either used alone [22] or in combination with venetoclax [70]. However, although CPT1A inhibitors were used in the 1970s as anti-anginal drugs, clinical translation of these findings is severely hampered by pronounced hepatotoxicity observed upon CPT1A inhibition in treated patients [71, 72]. As such, further research is required to fully understand the functional roles of FAO in leukemic cell survival and chemoresistance in order to identify more tolerable ways to target FAO.

## Fatty acid metabolism supports leukemia cell survival beyond energy provision

Apart from the role of FAs in energy production, FAs also govern the oxidative stress response, membrane fluidity and play an important role in the integrated stress response [73]. AML cells show decreased spare capacity in their respiratory chain and palmitate treatment resulted in pronounced toxicity through enhanced FAO and mitochondrial ROS production. This highlights the pleiotropic effects of modulating FAO in AML cells at least in vitro [74]. Lipid oxidative stress is predominantly associated with lipid peroxidation of PUFA which leads to cell death by ferroptosis [48]. This is a cell death mechanism involved in the response to both standard AML therapeutics like cytarabine [75, 76] and FLT3 inhibitors [76, 77], as well as emerging treatment strategies like imetelstat [11] and eprenetapopt [78]. As described earlier, PUFA-induced ferroptosis can be counteracted by increasing cellular MUFA levels, which have a protective role against palmitate mediated lipotoxicity [49]. Indeed, the primary desaturase responsible for MUFA production, SCD, is an independent prognostic factor in AML with increased production of unsaturated FA being linked to relapse. SCD inhibition demonstrates a pronounced antileukemic effect in a subset of AML models both in vitro and in vivo and increases sensitivity to conventional chemotherapeutic protocols based on anthracyclines and cytarabine [23]. This finding is especially translationally relevant because the SCD inhibitor MTI-301/SSI-4 is currently in a clinical trial for the treatment of metastatic or unresectable and refractory solid cancers [79]. Surprisingly, the toxicity associated with SCD inhibition is not solely driven by oxidative stress and ferroptosis, as reversing lipid peroxidation did not prevent cell death caused by SCD inhibition [23]. In contrast, SCD inhibition enhanced AML sensitivity to FLT3 inhibition by increasing lipid oxidative stress and activating the ferroptosis pathway [77] thus highlighting that the exact biological role of lipid mediators in AML is context dependent. Additionally, despite the expected role of PUFA in AML cell death induction via ferroptosis, high expression of the main PUFA producing desaturase FADS1 is negatively prognostic in AML [80, 81] and its inactivation results in cell cycle arrest, differentiation and cell death [81]. Furthermore, relapsed/refractory AML display an increase in FA desaturation and blocking FADS1 and FADS2 increases sensitivity to venetoclax-based regimens. One potential explanation for FA desaturation being a specific vulnerability of leukemic cells circles back to their dependency on FAO because unsaturated FA are the preferred FAO substrate [82]. Increased FA desaturation in leukemic cells can be additionally explained by the need for recycling NAD^+^/NADH [4]. Indeed FADS enzymes have been shown to provide a mechanism for NAD^+^ recycling, allowing cells to maintain active glycolysis, cellular redox status and viability when the cytosolic NAD^+^/NADH ratio is reduced for example as a result of ETC dysfunction [83]. Interestingly, NAD^+^ levels are increased in relapsed/refractory AML patients and coupled with an increase in nicotinamide metabolism results in resistance to venetoclax [84]. Finally, NAD+ salvaging enzyme nicotinamide phosphoribosyltransferase (NAMPT) is a selective vulnerability of LSCs. NAD+ depletion in response to NAMPT inhibition induces cell death by inhibiting SCD and disrupting the balance of SFA and MUFA leading to lipotoxicity [85]. Taken together, these findings indicate that AML survival and maintenance is closely associated with NAD^+^/NADH, which is tightly coupled to FA metabolism.

Lipid droplets are critical organelles involved in maintaining cellular lipid and fatty acid balance. By sequestering lipids within the cell, they regulate energy production through restricted FA availability and help reduce oxidative stress by limiting lipids available for peroxidation. Additionally, they serve as storage units of neutral lipids and phospholipids in cancer cells, which can be mobilized through lipolysis or lipophagy [86]. This enables them to compensate for specific lipid imbalances caused either by pathophysiological conditions or pharmacological interventions in cancer cells, such as reduced cellular levels of MUFAs [87]. Lipid droplets have recently attracted significant interest, particularly in AML [88], as a novel metabolic vulnerability linked to leukemic cell chemosensitivity and therapy resistance [89].

The role of FAS and FAO in modulating the oxido/reductive state of cells, explains why they have both been proposed as metabolic vulnerabilities in AML. However, a deeper understanding of FA metabolism interactions with redox cellular state is needed to define the best approach to target it therapeutically.

One important caveat when interpreting the results of studies of lipid and FA metabolism is that the great majority of them have been performed in vitro, often using classic culture conditions which poorly reproduce the physiological lipid composition of both the intracellular and extracellular milieu [90]. While the expanding use of more physiological media conditions might address some of these limitations, a greater understanding of lipid metabolism in vivo, taking into account leukemia cell interactions with the tumor microenvironment, is required to fully assess the functional role of FA metabolism pathways in leukemia establishment and progression. The adipocytic niche in particular plays a key role in modulating FA metabolism in normal and leukemic hematopoiesis. In the next section we will summarize current knowledge on how the adipocytic niche and specific adipocyte characteristics in the microenvironment affect normal and leukemia cell FA metabolism, cell fate decision and clinical responses to therapy.

## Bone marrow adipocyte distribution and cellular function: diversity and challenges

Adipocytic niches exist throughout the body and are diverse in both morphology and function. BM is the location of AML development and leukemic cell interaction with different BM cellular compartments plays pivotal roles in multiple types of drug resistance. While it has long been recognized that adult human bones are rich in adipocytes and that bone marrow adipocyte (BMAd) volume and density vary across different bones and regions, the functional significance and heterogeneity of the bone marrow adipose tissue (BMAT) niche has only more recently garnered attention [91]. A new and growing body of literature highlights important functional and metabolic differences between BMAd subtypes and their distinction from classical white adipocytes [92]. Elucidating these differences is essential for advancing our understanding of how different adipocyte niches influence AML disease progression and therapeutic resistance, particularly in the context of metabolism.

**Fig. 2:**
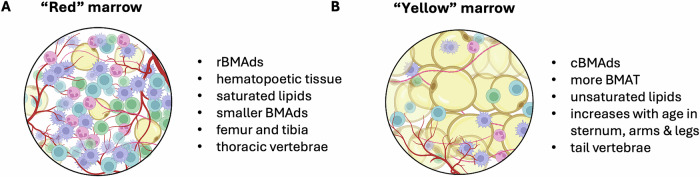
Types of bone marrow adipose tissues and interactions with hematopoietic cells. **A** Blood cell producing “red” marrow. **B** Adipocyte rich “yellow” marrow. Figure was generated using Biorender.com.

Functional differences have indeed been shown between the BMAT associated with blood producing “red” marrow adipose tissue (AT) and the “fatty” yellow marrow (Fig. 2). The red marrow AT is classified as regulatory BMAT (rBMAT) due to its responsiveness to external stimuli like fasting, exercise, and cold exposure while the yellow marrow AT, which remains unaffected by these stimuli, is classified as constitutive BMAT (cBMAT) [91, 93]. Furthermore, histological analyses have revealed differences in distribution and morphology between murine BMAds in rBMAT and cBMAT. rBMAds are smaller, associated with osteoblasts, endothelial cells, and hematopoietic cells, and are scattered throughout hematopoietic tissue. In contrast, cBMAds are larger and densely packed in highly adipocytic regions [91, 94]. Interestingly, rBMAds and cBMAds also differ in their gene expression and lipid composition. rBMAds contain more saturated lipids, while cBMAds are enriched with unsaturated lipids such as oleate and palmitoleate [95]. This observation is especially intriguing in the context of our findings that AML is functionally dependent on fatty acid desaturation [23], which makes it metabolically adapted to palmitate-rich surroundings typical for the “red” BM. Still, it is important to note that while murine cBMAds and rBMAds likely correspond to human counterparts, this remains unconfirmed [96].

Metabolic differences between BMAd subtypes imply the existence of distinct adipose BM metabolic niches. However, isolation and maintenance of adequate numbers of BMAds from different regions in both mice and humans is technically difficult, limiting the ability to study them as distinct biological entities. Currently, most functional studies on BMAds use in vitro models of murine or human adipocyte differentiation. While in vitro studies are important for better understanding of cellular interactions, such studies are unfortunately limited by their inherent lack of heterogeneity, negation of contextual based interactions and poor recapitulation of functions in situ [92]. Moreover, these studies are harder to interpret because it is unclear which BMAd subset is represented by the in vitro differentiated adipocytes. However, recent advancements in developing an in vitro model for rBMAds and cBMAds, along with improvements in isolating primary mature adipocytes, are promising and could help in further elucidation of BMAds functional dependencies [97].

## Influence of adipocytes on hematopoiesis

BMAds were initially proposed to negatively regulate the hematopoietic environment due to the observation that there are reduced numbers and frequencies of HSCs in the adipocyte rich tail vertebrae (comprising cBMAT) compared to adipocyte sparse thoracic vertebrae (comprising rBMAT) [98]. Furthermore, adipogenic lineage expansion during obesity and aging has been correlated with impaired stem cell regeneration and hematopoietic reconstitution [99]. In contrast, in vitro co-culture studies with human cells have demonstrated that adipocytes enhance hematopoiesis [100]. Furthermore, an increase in hematopoiesis and lymphocyte differentiation was correlated with increased BMAd numbers and elevated serum leptin levels in obese mice fed a high-fat diet (HFD) [101]. However, the immune and/or metabolic mechanisms behind these observations were not clearly identified. It is likely that variation arises through the association between adipose tissue and hematopoiesis being highly dependent on the physiological context and anatomical location, as well as the stage of adipocyte differentiation [102, 103]. Interactions between adipocytes and HSCs have been associated with the secreted cytokine milieu at each adipocyte development stage. Yet, we speculate that secreted metabolic mediators may also differ with adipocyte differentiation stage and influence HSC regulation. HSCs adapt their metabolism under different conditions. For example, in response to infection HSCs uptake long chain FAs via CD36 to fuel β-oxidation and drive proliferation [65]. While the source of the FAs and the influence of different BMAT regions remains unknown, this highlights how pathophysiological context may influence adipocyte and HSC interactions. Increased BM adiposity has also been correlated with higher density of maturing myeloid cells, and an increased proportion of HSCs adjacent to adipocytes may contribute to the expansion of pre-leukemic clones carrying mutations in genes recurrently mutated in AML such as *DNMT3A* [104]. While anatomical location and the role of specific adipocyte subsets has not been explored, these two lines of evidence suggest that adjacency to adipocytes in the BM microenvironment promotes myeloid skewing and expansion of aged (likely mutated) HSCs, contributing to age-related risk of myeloid malignancies [105].

## Influence of BMAds on AML and therapy resistance

**Fig. 3:**
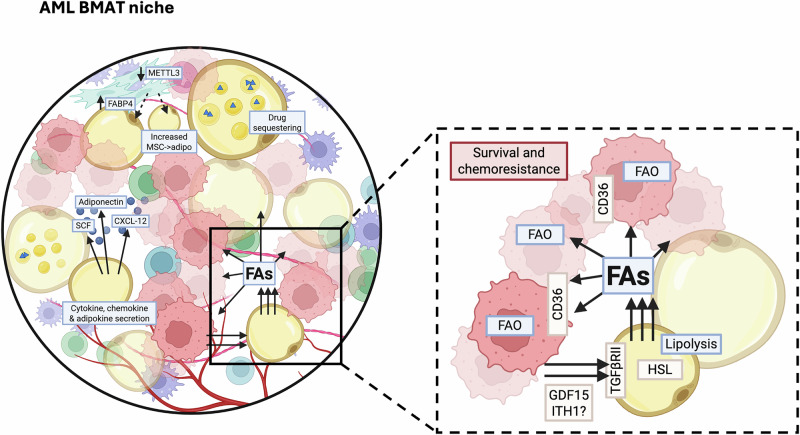
Interactions of AML cells and adipocytic niche. CD36 fatty acid transporter, CXCL12 C-X-C motif chemokine 12, FA fatty acids, FABP4 fatty acid binding protein 4, FAO fatty acid oxidation or β-oxidation, GDF15 Growth/differentiation factor 15, HSL hormone sensitive lipase, METTL3 N6-adenosine-methyltransferase 70 kDa subunit, MSC mesenchymal stromal cell, SCF stem cell factor, TGFβRII transforming growth factor β receptor II. Figure was generated using Biorender.com.

In solid cancers it is reported that adipocytes support survival and proliferation through release of adipokines, cytokines and chemokines [106, 107]. In AML, however, the most widely reported interaction between adipocytes and AML cells is the transfer of FAs to AML cells to fuel FAO and promote cell survival and chemoresistance [108–110]. It is increasingly reported that AML cells induce lipolysis of adipocyte triglyceride stores and import the released FAs through CD36 (Fig. 3) [111]. AML induces conversion of large to small adipocytes, a probable morphological sign of lipid and FA transfer from adipocyte to malignant cells, by releasing growth differentiation factor 15 (GDF15) [112]. GDF15 binding to TGFβ-RII on BMAds leads to the inhibition of transient receptor potential vanilloid 4 (TRPV4), which activates hormone sensitive lipase (HSL) resulting in lipolysis [113].

Reports about AML induced BMAd lipolysis are interesting considering that in physiologic conditions basal and induced lipolytic responses are reduced or even absent in human [92] and murine BMAds [93]. Proteomic and lipidomic analysis of BMAds and subcutaneous adipocytes isolated from patients undergoing hip replacement surgery revealed that the largest difference between BMAds and white adipocytes was in their lipid metabolism. Interestingly, BMAds showed pathway enrichment for cholesterol, lipoprotein and sphingolipid metabolism but relative downregulation of lipolysis regulation, FA and glucose metabolism. HSL and monoacylglycerol lipase (MGLL) were downregulated in BMAds and, when grown in vitro, these cells secreted undetectable levels of FA and glycerol both with and without stimulation with lipolytic activator isoprenaline [92]. Although primary isolated BMAds appear to lack lipolytic activity under physiological conditions, with lipolysis being activated to produce FA as an energy source for malignant cells only following AML-derived reprogramming, it is important to note that these observations were made in the absence of the in vivo microenvironment. Moreover, unlike primary isolated BMAds, adipocytes derived from BM-MSCs and OP9 cells displayed lipolytic activity upon lipolysis stimulation in vitro [92] thus again highlighting issues associated with in vitro adipocyte differentiation. Interestingly, studies in chronic myeloid leukemia in blast crisis have shown that adipose triglyceride lipase (ATGL), but not HSL or MGLL, is upregulated in gonadal adipose tissue, which functions as a leukemic stem cell niche. This suggests the possibility of conditional lipase specificity [114]. Furthermore, relatively higher numbers of small adipocytes in patients correlate with increased risk of relapse and shorter overall survival [115]. As already mentioned, it is likely that the reduction of adipocyte size is associated with increased lipolysis thus a consequence of the lipolytic benefits AML cells obtain [112]. Taken together, this supports the notion that BMAd lipolytic activity is context and adipocyte subtype dependent and might be a targetable dependency in AML.

Furthermore, observations that AML mesenchymal stromal cells (MSCs) display increased adipogenic potential compared to healthy MSCs also indicate BMAd importance to AML cells. Increased adipogenic potential has been associated with SOX9 dependent mechanisms [116], upregulation of fatty acid transporter FABP4 [117] and downregulated METTL3 expression (Fig. 3) [118]. However, as with many BMAd studies, other studies demonstrate the opposite. As such, inhibited adipogenesis and increased MSC osteogenic differentiation have also been reported in AML [119]. Unsurprisingly, the influence of AML on BMAd number is again context dependent. Study of human trephines and AML xenografts revealed that AML reduced the absolute number and the size of BMAds in areas of red marrow but not yellow marrow, despite AML infiltration at all bone sites [120].

Beyond the secretion of mediators, adipose tissue is associated with drug metabolism and inactivation through sequestration in lipid droplets (Fig. 3). Concordantly, human and murine adipose tissue actively metabolize daunorubicin thereby reducing concentrations in the local environment and contributing to chemoresistance in acute lymphoblastic leukemia models [121, 122]. Similar work has not been conducted in AML, but it can be assumed that this pharmacokinetic mechanism would remain consistent. It is also important to consider the influences that AML therapies have on the AML BMAd niche. While it is shown that irradiation and chemotherapy increase BMAd content in solid cancer patients [123], the effect of targeted therapies on the BMAd niche in AML has not been explored. However, with increasing data highlighting how BMAds promote AML survival and drug resistance, understanding therapy induced changes in BMAd niches and associated lipid metabolism will be required for successful targeting of BMAd-induced drug resistance mechanisms.

## How to hijack AML connection with fats for more successful therapy?

AML cells co-opt lipid metabolism to withstand both environmental stress and treatment pressure. This, in turn, generates therapeutically actionable dependencies as AML cells need to tightly control their FA levels because both SFA and PUFA in excess are toxic due to increased endoplasmic-reticulum stress and lipid peroxidation respectively. Co-opting FA metabolism also renders AML cells reliant on FAO which both utilizes FA as an energy source and reduces their toxic build-up. Therefore, many facets of lipid metabolism have been explored as targets for the development of new therapeutic strategies in AML. These, however, have so far not translated to the clinic effectively either because of toxicity and/or lack of efficacy. There are several reasons for this, including an incomplete understanding of the mechanisms through which FA metabolism supports cell survival and therapy resistance, significant intra- and inter-tumor metabolic heterogeneity and limited knowledge of microenvironmental rescue. Therefore, to realize the full translational potential of targeting lipid metabolism in AML, it is essential to better define the cellular and metabolic contexts in which specific therapeutic vulnerabilities are generated. This will require identification of cell intrinsic mutational, transcriptional or metabolic markers of sensitivity to specific FA metabolism inhibitors. Moreover, implementing the use of growth media closely mimicking physiologic levels of nutrients in peripheral blood, bone marrow or extramedullary sites such as cerebrospinal fluid is also needed. Additionally, improved modeling of AML microenvironments in vitro — ideally in 3D systems that more accurately reflect in vivo conditions — will help in avoiding many of the pitfalls currently encountered when translating in vitro findings into more complex model systems or to the clinic. This will allow for a deeper understanding of the micro- and macroenvironmental modulators of AML cellular dependencies on FA metabolism, in particular, the role of the adipose tissue niche, given its prominent role in modulating the FA metabolism of both normal hematopoietic and leukemic cells. Only a detailed understanding of the cell intrinsic and extrinsic factors modulating FA metabolic adaptation and dependencies in AML cells can lead to its optimal therapeutic targeting and fulfill the promise of targeting lipid metabolism as an effective AML therapy.
